# Human papilloma virus (HPV) genotypes concordance between Iranian couples referrals

**DOI:** 10.1186/s13027-019-0241-x

**Published:** 2019-09-09

**Authors:** Mehrdad Davarmanesh, Mehrouz Dezfulian, Mohammad Javad Gharavi, Sarang Younesi, Pourandokht Saadati, Mohammad Mehdi Taheri Amin, Seyed Mohammad Jazayeri

**Affiliations:** 1 Department of Microbiology, Karaj Branch, Islamic Azad University, Karaj, Iran; 2 Faculty of Paramedicine, Department of Laboratory sciences, Iran University of Medical Sciences, Tehran, Iran; 3 Nilou Medical Laboratory, Tehran, Iran; 4 Research Center for Clinical Virology, Tehran University of Medical Sciences, Tehran, Iran; 5 Medical Genetic Laboratory, Laleh Hospital, Tehran, Iran

**Keywords:** Human papilloma virus (HPV) genotypes, HPV genotypes concordance, Cervical cancer

## Abstract

**Background:**

Human Papilloma Virus (HPV) genotypes concordance among sexual couples has been evaluated in many investigations with considerable variations in the concordance. However, no such study has carried out between Iranian couples yet**.**

**Methods:**

Urogenital specimen from both males and females of couples were taken and transferred to Nilou laboratory for molecular analysis. HPV DNA extraction and typing were carried out using cobas 4800 platform. Demographic and virological data were analyzed afterwards.

**Results:**

One hundred fourteen couples were enrolled in the study. The mean age of participants were 36 ± 8 and 32 ± 7 for males and females, respectively. 64 (28%) of specimens were positive for at least one HPV genotype. The positive rates within genders were 30.7 and 25.4% for females and males, respectively with a considerable association (*P* value 0.021). Within the positive samples, 13(5.7%), 8 (7%) and 31(13.5%) were belonged to 16, 18 and other HR genotypes. 59 (51.8%) couples who were negative for HPV showed negative concordance. Of the total positive HPV patients (55 couples, 48.2%), 9 (16.3%) couples had positive concordance and the rest of 46 (83.7%) couples (either of spouse being negative and the other being positive for HPV) showed neither kinds of concordance.

**Conclusion:**

Recognition of the dynamics of HPV infection not only in women, but in their sexual partners could impact the implementation of preventive measures like HPV vaccination for cervical cancer and other HPV-related diseases for both sexual partners.

## Background

Worldwide, HPV infection accounts for an estimated 530,000 cervical cancer cases as well as 270,000 deaths annually occurring substantially in developing countries [1]. HPV also is the fundamental vehicle of vulvar and vaginal cancer in women and penile cancer in men. It is estimated that 40% of penile carcinomas are attributed to infection with high risk HPV [2–4] and approximately, 20% of all men (reaching 70% in some age groups), especially among individuals between 15 and 24 years of age are infected with HPV-infection [1]. Sexual behavior characteristics in both women and men are key determinants of HPV infection, worldwide. Moreover, previous reports confirmed that the sexual behavior of males can influence the risk of cervical cancer in their sexual partners [4–7]. The detected prevalence of HPV infection in male partners of women who were positive for HPV and/or cervical intraepithelial neoplasia or squamous carcinoma, ranged between 23 and 73% in several reports [4–6, 8]. Although compared to women, HPV infection may be associated with lower mortality and morbidity in men, however, due to its association with genital warts, penile, anorectal and oropharyngeal cancers as well as to the risk of HPV transmission to their female sexual partners research in this field remains essential [9–11] Accordingly, females partners of men having penile cancer showed cervical cancer prevalence eight times higher, whereas male partners of women having cervical cancer showed higher risk of developing penile cancer [12]. Transmission can occur easily between sexual partners, and in many cases, multiple transmission incidence may occur within a couple without being detected in either partner [5, 6, 13, 14]. The HPV type concordance between sex partners has been investigated in previous reports although with evident variations in the concordance [15–18]. Substantial variations in HPV type concordance are obvious, which may be explained by differences in the variety of HPV types examined, the different methodologies used for sampling (especial for men) and the population studied. Moreover, some investigations have recognized that HPV type concordance may be related to the concentration of viral DNA [15]. Evidence about HPV prevalence and its concordance in couples is of supreme importance for the evaluation of the impact of prophylactic vaccines against HPV and to monitor the spreading of specific HPV types before and after the introduction of HPV vaccines in populations. However, no investigation has evaluated the concordance of HPV types between couples or the prevalence of HPV infection in sexual partners of women in Iran.

The aims of the present survey were to determine HPV prevalence in heterosexual couples and to evaluate HPV type-specific concordance in a predominantly monogamous population.

## Methods

### Study population and specimen collection

**Fig. 1:**
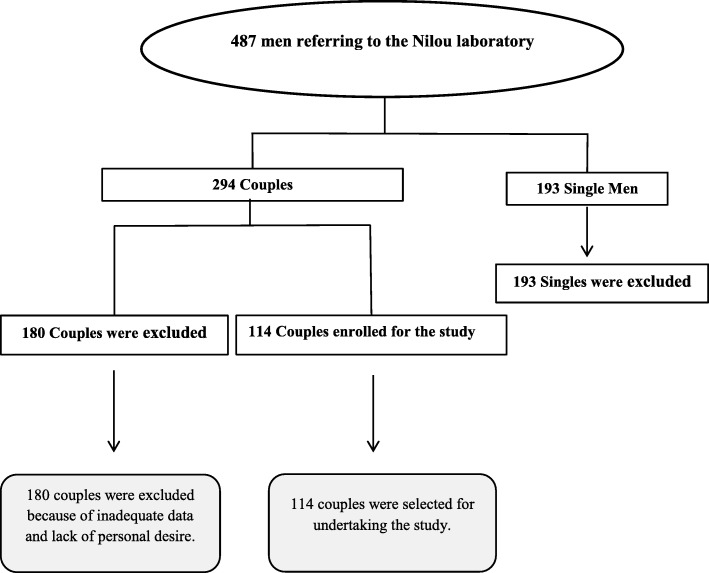
An algorithm showing participants recruitment in the survey

This study was a longitudinal, cross sectional investigation on the prevalence of type-specific HPV concordance in sexually active couples during 2017–2018. To recognize the couples, men files were reviewed firstly. This was due to the fact that women consisting a majority of referrals to Nilou laboratory and finding couples through female files was troublesome. The inclusion criteria were a steady female partner (at least for the last 6 months even if they were not living in the same house) and, if so, whether they would invite their partner to join the couples’ survey. According to the Fig. 1, 487 men’s files were registered for HPV typing in Nilou Genetic Laboratory during 2017–2018. One hundred ninety-three single men were excluded. Also, 180 couples were dropped out after successfully meeting the inclusion criteria due to unwillingness to participation. Thus, a total of 114 sex partner participants were enrolled in the current investigation (Fig. 1).

A written informed consent guaranteeing confidentiality was taken from each patient. The study was approved by the National Ethics Committee for Biomedical Research affiliated to The Ministry of Health and Medical Education. According to HPV testing results, couples categorized into: positive concordance (both partners sharing the same HPV genotype), negative concordance (both being negative for HPV) and neither of discordance (when either of spouse being positive and the other being negative for HPV).

### Sampling

The epithelial cells from the urinary meatus, frenulum of prepuce and glance of penis were obtained using a cytobrush, this was cut and inserted in *thinprep* tubes and tested immediately. For women, cervical scrapes were collected by sampling the ectocervix and endocervix with a cytobrush and cervical scrapes were also inserted in a tube containing lysis buffer. Alternatively, cervical samples were taken by a gynecologist from the endocervix and ectocervix areas with a sterile cytobrush and subsequently transferred to Nilou laboratory.

### HPV testing

The cobas 4800 HPV test included fully automated sample preparation combined with real-time PCR technology and software that integrates the two modules. One-milliliter aliquots of thinprep fluid were transferred to 13-mL barcoded tubes provided by the manufacturer. The cobas 4800 HPV test was performed according to the manufacturer’s protocol (Roche Molecular Systems, CA, USA). COBAS generates individual qualitative results for HPV 16, HPV 18 and a pool of other high risk HPV genotypes.

### Statistics

Statistical analysis was performed using SPSS software version 22 package. Cramer’s V test was used to for correlation intensity between HPV genotypes in couples. This test showed distribution of categorical data in different groups in this study. The concordance analysis was based on the couples’ enrollment data. The proportion of couples who were concordant and discordant was calculated. A couple was classified as having “type-specific positive concordance” if the man and woman had ≥1 HPV genotype in common. A couple was classified as having “negative concordance” if both the man and woman were negative for 14 high risk genotypes. *P* value was considered significant when < 0.05.

## Results

**Fig. 2:**
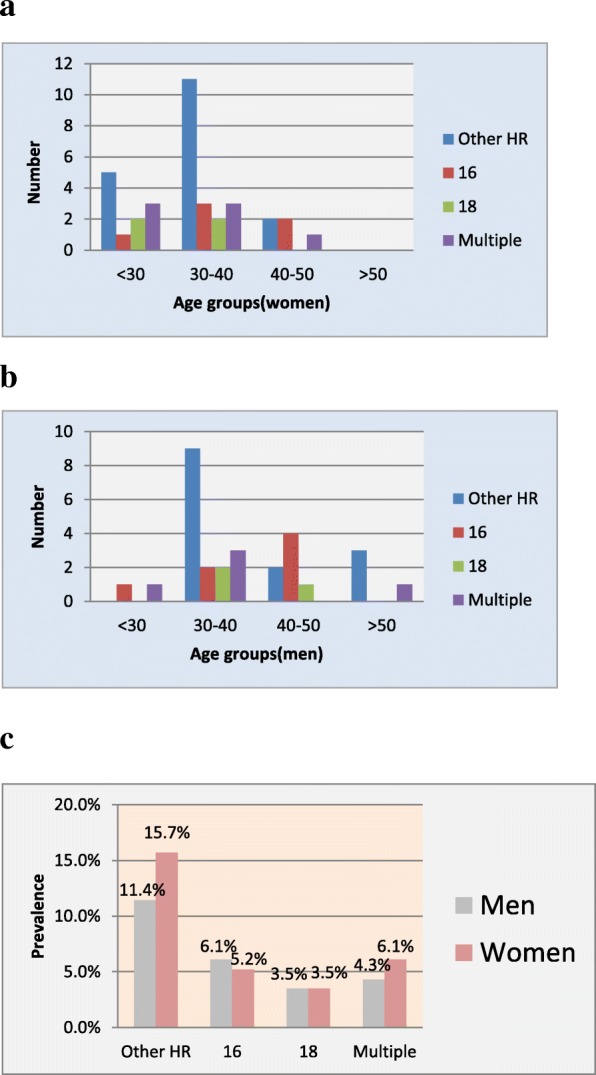
Distribution of positive HPV genotypes. **a** HPV genotypes distribution according to age groups in women (**b**) and men (**c**) and according to genders

Out of 487 men referred to Nilou laboratory, 193 were single without a clear history of sextual partnership and hence excluded from the survey. Finally, 114 couples were eligible and were enrolled in this study (Fig. 1). Table 1 shows the demographic and details of HPV assay analysis of individuals. The mean age of participants were 36 ± 8 and 32 ± 7 for males and females, respectively without any significant association between genders among different age groups (Table 1). Considering the different age groups, in both genders, the highest prevalence was found between age group of 30 and 40 years old (48.6% of total population), however, with no significant associations (Table 1, Fig. 2, a and b). 64 (28%) of specimens were positive for at least one HPV genotype which were consisted of 35 (30.7%) and 29 (25.4%) for females and males, respectively (*P* value, 0.021, Table 1). 52 (81.2%) and 12 (18.8%) of positive samples were composed of single and multiple HPV genotypes respectively (*P* value 0.433, Table 1). Within the positive samples, 13(5.7%), 8 (7%) and 31(13.5%) were belonged to 16, 18 and other HR genotypes (Table 1). Between females, 6 (5.2%), 4 (3.5%) and 18 (15.7%) and among males, 7(6.1%), 4 (3.5%) and 13 (11.4%) were positive for 16, 18 and other HR genotypes, respectively (Table 1). Among genders, other HR genotypes than 16 and 18 prevalence were reached statistically significant (*P* value, 0.047, Table 1, Fig. 2, c).

**Table 1:** Demographic and HPV genotypic characteristics of patients

Characteristic	All samples	Men	Women	*P* value	CI 95%
	n(%)	n(%)	n(%)		(for Age and prevalence of infection)
	228(100)	114(100)	114(100)	α =0.05	Lower - Upper
Age					
<30	69(30.2)	23(20)	45(39.4)	0.279	23.7–36%
30–40	111(48.6)	58(50.8)	53(46.4)	0.789	42.1–55.7%
40–50	40(17.5)	27(23.6)	13(11.4)	0.570	12.7–22.4%
>50	9(3.9)	6(5.2)	3(2.6)	0.325	1.8–6.6%
HPV Genotype (High Risk)					
Negative	164(71.9)	85(74.5)	79(69.2)	0.084	65.8–78.1%
Positive (Total)	64(28)	29(25.4)	35(30.7)	0.021	21.9–34.2%
16	13(5.7)	7(6.1)	6(5.2)	0.301	3.1–8.8%
18	8(7)	4(3.5)	4(3.5)	0.202	1.3–6.1%
Other HR Genotypes	31(13.5)	13(11.4)	18(15.7)	0.047	9.2–18%
Multiple^a^	12(5.2)	5(4.3)	7(6.1)	0.433	2.6–8.3%

^a^16&Other HR, 18& Other HR,16&18

Regarding the couples, they were classified into four groups. Groups I consisted of 59 (51.8%) couples who were negative for HPV,whereas, groups II, III and IV comprised the total positive HPV patients (55 couples, 48.2%) (Table 2). Among group II, all couples showed positive concordant, but only one husband showed partial concordance who had HPV-16 along with other HPV genotypes whereas his wife had only other types (Table 2, group II). Group III was composed of 20 couples (17.5%) in whom females were negative for HPV; however, their male partners were positive for at least one HPV genotype (Table 2). This group had five HPV-16, four HPV-18, two 16+ others and nine others HPV genotypes. Females who were positive, but their partners were negative for HPV were belonged to group IV, 26 couples (22.8%, Table 2). This group had four HPV-16, four HPV-18, three mixed 16 and 18, two 16+ others, one 18+ others and twelve others HPV genotypes. In total, of groups all groups studied, group I considered as being negative concordance; group II, positive concordance and groups III and IV showed neither kinds of concordance.The highest rate of HPV discordant results was observed for group IV (Table 2).

**Table 2:** The characteristics of HPV types according to different groups in the couples-studied

HPV detection: Men ± / Women ±	Men (Genotype)	Women (Genotype)
Group I: Men**– /** Women**–**(*n* = 59)	Neg	Neg
Group II: Men**+ /** Women **+** (*n* = 9)	Other HR	Other HR
	Other HR	Other HR
	Other HR	Other HR
	Other HR	Other HR
	Other HR	Other HR
	16&Other HR	Other HR
	16&Other HR	16&Other HR
	16	16
	16	16
Group III: Men **+/** Women **–** (*n* = 20)	Other HR	Neg
	Other HR	Neg
	Other HR	Neg
	Other HR	Neg
	Other HR	Neg
	Other HR	Neg
	Other HR	Neg
	Other HR	Neg
	Other HR	Neg
	16& Other HR	Neg
	16& Other HR	Neg
	16	Neg
	16	Neg
	16	Neg
	16	Neg
	16	Neg
	18	Neg
	18	Neg
	18	Neg
	18	Neg
Group IV: Men**– /** Women**+** (*n* = 26)	Neg	Other HR
	Neg	Other HR
	Neg	Other HR
	Neg	Other HR
	Neg	Other HR
	Neg	Other HR
	Neg	Other HR
	Neg	Other HR
	Neg	Other HR
	Neg	Other HR
	Neg	Other HR
	Neg	Other HR
	Neg	16& Other HR
	Neg	16& Other HR
	Neg	18& Other HR
	Neg	16&18
	Neg	16&18
	Neg	16&18
	Neg	16
	Neg	16
	Neg	16
	Neg	16
	Neg	18
	Neg	18
	Neg	18
	Neg	18

## Discussion

HPV infection is very widespread among males and females across all geographical, racial and socio-economic populations worldwide and is the only sexually transmitted disease that is difficult to be handled in both members of a sexual couple. The substantial increased prevalence of genital tract HPV infections in many regions has been ascribed to an early start of sexual activity, poor sexual hygiene, multitude number of sexual partners, and insufficient preventive measures [19]. The objectives of the current investigation were to characterize the type-specific HPV genital infection positivity distribution and and to analyse their concordance in a group of stable heterosexual partners who referred to Nilou laboratory by gynecologists.

HPV genotypes prevalence and its concordance among heterosexual couples have been studied in many trials although with evident variations in the concordance. However, there are not too many reports on HPV concordance between referral subjects [20–22] (Table 3). General and type-specific HPV infection concordance in sexual partners have been assessed in these surveys with heterogeneous results which could be explained by the target populations, the DNA detection techniques and sampling methods-used (Table 3). Even two reports from the same countries (the USA and mexico) showed completely different results (Table 3). The range of type-specific HPV positive concordance is wide, ranging from as low as 16.6 to 59% (Table 3) suggesting that concordance is more variable than expected by chance. Present findings revealed that only 9 (16.3%) couples had positive concordance, which was showed the lowest rate among surveys on HPV compliance between referral couples outlined in Table 3. Our study along with Prada’s report from Mexico [20] showed the lowest proportion of positive concordance between couples (16.3% and 16.6%, respectively, Table 3).

**Table 3:** Reported concordance of HPV genotypes between referral couples among different surveys

Author/Year	Country	Men HPV + (%)	Women HPV + (%)	HPV genotype Concordance	
				Negative Concordance (%)	Positive Concordance (%)
Nyitria et al. [21]/ 2012	USA	55.6	45.4	63.1	36.8
Vargas et al. [23]/2016	Colombia	56	80	68	31
Parada et al. [20]/2010	Mexico	20.4	13.7	83.2	16.6
Hernández-Sotelo et al. [24]/2016	Mexico	100	92	67.8	32.1
Widdice et al. [22]/2010	USA	76	84	44	56
Present Study/2019	Iran	25.4	30.7	83.6	16.3

There are almost no uniform findings between HPV genotypes concordance between sexual partners. They seem to depend upon the type of sexual relationship between male and female and the duration of relationship between couples, immune status differences between them and possibly other environmental factors [6, 13]. Immune responses may affect the viral load, the alternation of viral types, and therefore, discordance between partners with long-term relationships [1]. Moreover, the lack of concordance in a proportion of couples may be explained by differences in the time required for elimination of HPV infection in males and females.

The prevalence of HPV genotypes in couples differs among different surveys, reporting rates of 3.5 to 59% for HPV 16, from 3.5 to 6.7% for HPV 18, and from 3.5% to 8.5% for other HPV genotypes [3, 17, 23]. Types 16 and 18 are responsible for at least 70% of cervical cancer incidences and especially HPV 16 accounts for a large proportion of other cancers attributed to HPV in males and females. Moreover, types 16 and 18 appear to be more persistent than other HPV genotypes and therefore differences between viral genotypes in terms of clearance time may also influence concordance between couples [24–28].

Admittedly, there are some shortages for this study. Firstly, small sample size prevents generalization of results to the country. Secondly, we did not evaluate the existing risk factors due to unwillingness of participated partners. Evaluation of these factors associated with type-specific positive concordance in healthy heterosexual couples is necessary to increase our understanding of HPV acquisition and transmission dynamics [13]. Thirdly, no monitoring of the duration and clearance of the HPV infection among couples was carried out.

In conclusion, it is still important to investigate the HPV genotypes concordance between couples. Recognition of the dynamics of HPV infection not only in women, but in sexual partners could impact the implementation of preventive measures for cervical cancer and other HPV-related diseases. Protective measures like HPV vaccination in men will protect not only them but will also benefit their sexual partners. Therefore, policies to mitigate HPV infections in couples may give rise in public health improvement.

## Data Availability

Authors can confirm that all relevant data are included in the article and materials are available on request from the authors.
